# Otorrhagia during robotic colorectal resection: a two case series and review of the literature

**DOI:** 10.1093/jscr/rjag404

**Published:** 2026-05-27

**Authors:** Michał Kazanowski, Paweł Lesiak, Bartosz Kapturkiewicz, Jędrzej Wierzbicki, Paweł Maciejewski, Marek Bębenek

**Affiliations:** Department of Surgical Oncology, Lower Silesian Center of Oncology, Pulmonology and Hematology, Wroclaw, Poland; Department of Surgical Oncology, Lower Silesian Center of Oncology, Pulmonology and Hematology, Wroclaw, Poland; Department of Surgical Oncology, Lower Silesian Center of Oncology, Pulmonology and Hematology, Wroclaw, Poland; Department of Surgical Oncology, Lower Silesian Center of Oncology, Pulmonology and Hematology, Wroclaw, Poland; Laboratory of Immunopathology, Department of Experimental Therapy, Hirszfeld Institute of Immunology & Experimental Therapy; Polish Academy of Sciences, Wroclaw, Poland; Department of Surgical Oncology, Lower Silesian Center of Oncology, Pulmonology and Hematology, Wroclaw, Poland; Department of Surgical Oncology, Lower Silesian Center of Oncology, Pulmonology and Hematology, Wroclaw, Poland; Faculty of Medicine, Wroclaw University of Science and Technology, Wroclaw, Poland

**Keywords:** otorrhagia, robotic colorectal surgery, Trendelenburg position, tympanic membrane injury, pneumoperitoneum

## Abstract

Perioperative otorrhagia is a rare complication of laparoscopic and robotic pelvic surgery performed in Trendelenburg position with pneumoperitoneum. We report two cases occurring during robotic colorectal resection performed in 25-degree Trendelenburg with 12 mmHg pneumoperitoneum. A 74 year old woman undergoing robotic sigmoid resection developed left sided otorrhagia intraoperatively, with computed tomography and otolaryngologic examination demonstrating blood in the external auditory canal and tympanic membrane injury. A 63 year old man undergoing robotic low anterior resection developed bilateral otorrhagia during emergence from anesthesia. Both patients were managed conservatively with otolaryngologic follow-up and recovered without ongoing bleeding or subjective hearing impairment. Previously reported cases have been described mainly in urologic and gynecologic surgery, usually under steeper Trendelenburg conditions. These cases show that perioperative otorrhagia may also occur during robotic colorectal surgery under moderate positioning parameters and is usually associated with a favorable outcome.

## Introduction

Perioperative otorrhagia is a rare complication of non-otologic surgery that has been described after laparoscopic and robotic pelvic procedures performed in Trendelenburg position with pneumoperitoneum [1–4]. The first reported cases were published in 2001 after laparoscopic gynecologic surgery, and subsequent reports have arisen predominantly from urologic and gynecologic procedures, with only isolated colorectal cases in the literature [1–4]. Reported otologic findings have included external auditory canal hematoma, intratympanic hematoma, hemotympanum, and tympanic membrane perforation [1–4].

The underlying mechanism remains incompletely understood. Proposed explanations include cephalad venous congestion related to head down positioning, increased pressure within the external or middle ear during pneumoperitoneum, impaired Eustachian tube function under general anesthesia, and barotrauma associated with prolonged pelvic surgery [3–5]. Most published cases occurred during procedures performed in steep Trendelenburg position, although reports in reduced Trendelenburg and lower pressure pneumoperitoneum suggest a multifactorial etiology and possible patient specific susceptibility [2–5].

We present two cases of perioperative otorrhagia during robotic colorectal resection performed in 25-degree Trendelenburg with 12 mmHg pneumoperitoneum, further supporting that this uncommon complication may occur even under moderate positioning conditions.

## Case series

**Figure 1:**
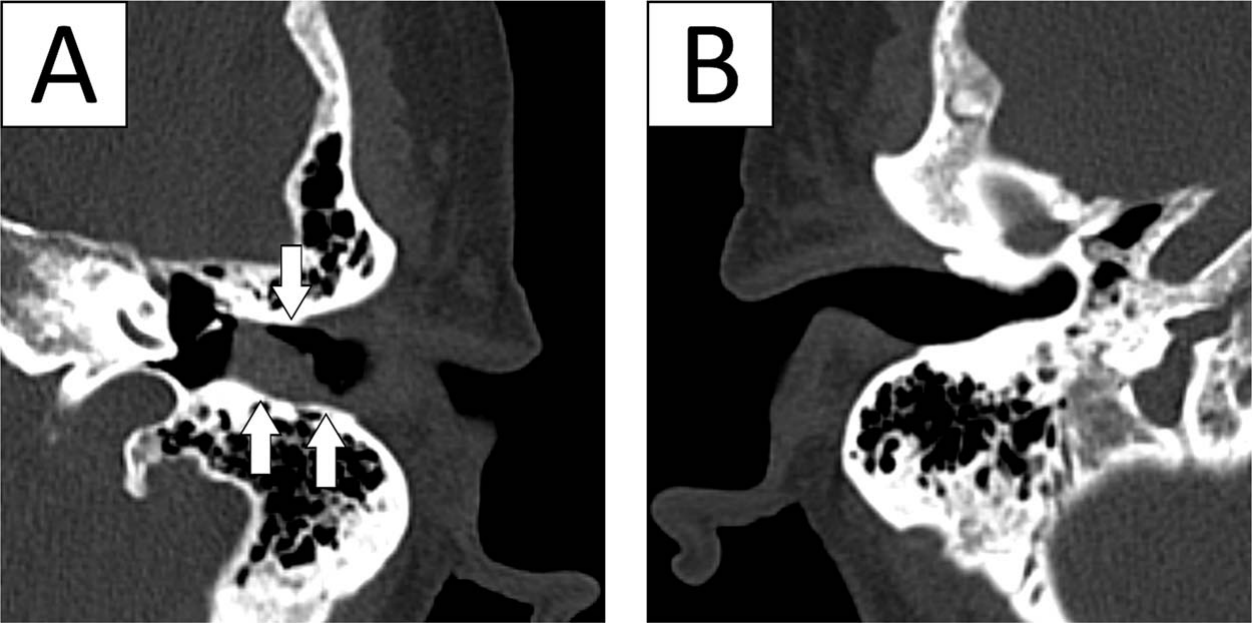
Non-contrast head CT in case 1. (A) Affected left ear demonstrating hyperdense blood within the external auditory canal at the level of the tympanic membrane, with adjacent soft-tissue blood products near the auricular/mastoid region (arrows). (B) Contralateral non-affected ear without comparable abnormality.

**Figure 2:**
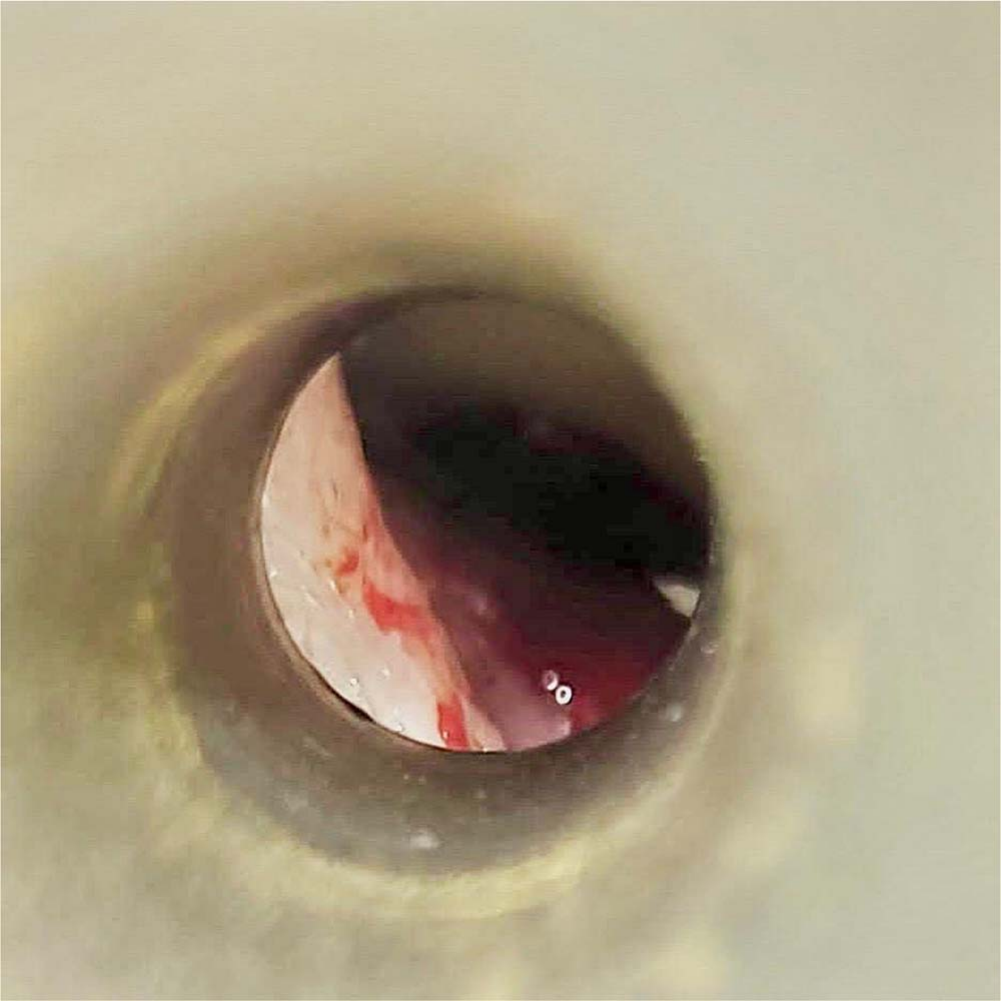
Otolaryngologic image from case 1 demonstrating injury of the left tympanic membrane with fresh blood in the external auditory canal.

A 74 year old woman (BMI 24.0 kg/m^2^, ASA III) underwent robotic sigmoid resection for a sigmoid polyp not amenable to endoscopic resection. Her medical history included rheumatoid arthritis, diabetes mellitus, atrial fibrillation, and previous hysterectomy, thyroidectomy, bilateral knee arthroplasty, and hip arthroplasty. She had no prior otologic history. Chronic anticoagulation for atrial fibrillation had been discontinued 5 days before surgery. Standard thromboembolic prophylaxis with enoxaparin 40 mg was administered 12 hours preoperatively. The procedure was performed in 25-degree Trendelenburg with pneumoperitoneum pressure of 12 mmHg and lasted 205 minutes, corresponding to an estimated Trendelenburg time of ~185–190 minutes. No marked intraoperative blood pressure elevations were recorded. Approximately 45–60 minutes after robotic docking, active bleeding from the left ear was noted. Urgent non-contrast head computed tomography (CT) demonstrated hyperdense blood within the left external auditory canal at the level of the tympanic membrane, with a small amount of subcutaneous blood near the base of the left auricle and mastoid region; no intracranial hemorrhage or skull fracture was identified (Fig. 1). Intraoperative otolaryngology examination showed a macerated tympanic membrane with fresh bleeding from its inferior portion (Fig. 2), and gelatin sponge (Spongostan) was placed in the external auditory canal. Serial otorhinolaryngology / ear, nose and throat (ENT) follow-up showed gradual cessation of bleeding with repeated Spongostan application. The patient was treated with systemic antibiotics for 14 days and hydrogen peroxide drops 2-3 times daily, and was discharged on postoperative day 8 without further active bleeding or subjective hearing impairment.

A 63 year old man (BMI 22.3 kg/m^2^, ASA II) underwent robotic low anterior resection for cT2N0M0 rectal cancer without neoadjuvant treatment. His medical history included gout, previous lumbar vertebral prosthesis implantation, and appendectomy. He had no prior otologic history. Standard thromboembolic prophylaxis with enoxaparin 40 mg was administered 12 hours preoperatively. The procedure was performed in 25-degree Trendelenburg with pneumoperitoneum pressure of 12 mmHg and lasted 290 minutes, corresponding to an estimated Trendelenburg time of ~270–275 minutes. No marked intraoperative blood pressure elevations were recorded. Bilateral ear bleeding was noted at the end of the procedure during emergence from anesthesia. Non-contrast head CT showed no acute intracranial abnormality; incidental polypoid mucosal thickening was described in the frontal and maxillary sinuses. ENT assessment on the following day demonstrated a large amount of clotted blood in both external auditory canals, and the tympanic membranes could not be assessed because of blood. The patient was advised to keep both ears dry and was treated with systemic antibiotics for 14 days and hydrogen peroxide 3% three times daily, with Spongostan recommended in the event of recurrent fresh bleeding. He was discharged on postoperative day 5 with resolving bilateral otorrhagia and no subjective hearing complaints.

The main clinical, perioperative, and outcome characteristics of both cases are summarized in Table 1.

**Table 1:** Summary of case characteristics and outcomes

**Parameter**	**Case 1**	**Case 2**
Age/Sex	74/F	63/M
ASA	III	II
Procedure	Robotic sigmoid resection	Robotic low anterior resection
Trendelenburg	25°	25°
Pneumoperitoneum	12 mmHg	12 mmHg
Operative time	205 min	290 min
Estimated Trendelenburg time	~185-190 min	~270-275 min
Timing of bleeding	45-60 min after docking	End of procedure / emergence
Laterality	Left-sided	Bilateral
Tympanic membrane finding	Injury confirmed	Not assessable due to blood
Length of stay	8 days	5 days
Hearing outcome	No subjective hearing impairment	No subjective hearing complaints

## Discussion

Perioperative otorrhagia associated with Trendelenburg positioning and pneumoperitoneum is rare and has been reported mainly in isolated case reports and very small series [1, 3, 4, 6, 7]. Most published cases have involved robotic prostatectomy or gynecologic surgery, whereas colorectal reports are uncommon [2–4, 6–8]. In the limited colorectal literature, previously described cases occurred during laparoscopic procedures performed in steeper Trendelenburg positions, typically around 35–40 degrees [2, 8]. Our cases extend these observations by showing that otorrhagia may also occur during robotic colorectal resection performed in 25-degree Trendelenburg with 12 mmHg pneumoperitoneum.

The mechanism is likely multifactorial [3, 5, 9]. Trendelenburg positioning and pneumoperitoneum increase venous pressure in the head and neck and may promote vascular congestion within the external and middle ear [3, 5]. At the same time, pressure equalization across the tympanic membrane may be impaired under general anesthesia because normal Eustachian tube opening during swallowing is suppressed [9]. Together, these effects may predispose to external auditory canal bleeding, hemotympanum, or tympanic membrane perforation, depending on individual anatomy and the balance between pressure load and tissue tolerance [2, 5, 9]. Published reports illustrate this spectrum well, ranging from external auditory canal hematoma or sloughed epithelium with intact tympanic membranes to definite tympanic membrane perforation [4, 6, 7, 9].

A physiologic basis for this complication is supported by previous literature. A prospective study demonstrated that middle-ear pressure increases during robotic prostatectomy in steep Trendelenburg with pneumoperitoneum, with changes related to end tidal CO₂ and airway pressures [5]. Although most reported cases occurred during steeper positioning, the case reported by Aloisi et al. in reduced Trendelenburg and low pressure pneumoperitoneum, together with our two cases at 25 degrees, suggests that angle alone does not fully explain the event [3]. Duration of exposure, hemodynamic transitions during desufflation or repositioning, and patient specific susceptibility are also likely to be important. This interpretation is consistent with Addison et al., in which bleeding became evident after return to the supine position, and with our second case, in which bilateral bleeding was recognized during emergence from anesthesia [2, 3].

Our cases suggest that moderate positioning parameters do not eliminate risk. In case 1, older age, multiple comorbidities, and prior chronic anticoagulation may have lowered the threshold for clinically apparent bleeding, although therapeutic anticoagulation had been discontinued 5 days before surgery. In both patients, standard thromboprophylaxis with enoxaparin had been administered and no marked intraoperative hypertensive peaks were documented. However, the estimated duration of Trendelenburg remained substantial, at ~185-190 minutes in case 1 and 270-275 minutes in case 2. Taken together, these observations support the interpretation that prolonged cumulative physiologic stress may be at least as relevant as the absolute angle of tilt.

Management in the published literature and in our patients is predominantly conservative [4, 6, 7, 9, 10]. Once otorrhagia is recognized, otologic examination is important to distinguish canal bleeding from tympanic membrane injury and to exclude obvious traumatic causes. ENT consultation is useful, particularly when bleeding is active or the tympanic membrane cannot be assessed [4, 10]. In selected cases, imaging may be appropriate to exclude skull base injury or intracranial pathology when the presentation is unexpected or when neurologic concern exists. Most reported patients, including ours, were treated with local hemostatic measures, dry ear precautions, and topical and/or systemic therapy as clinically indicated [4, 6, 7, 9, 10]. Outcomes are generally favorable, with resolution and no permanent sequelae reported in most cases, although transient hearing symptoms may occur [4, 6, 7, 9, 10].

The practical implication is not that otorrhagia should alter routine selection of robotic colorectal surgery, but that surgeons and anesthesiologists should remain aware of it during prolonged pelvic procedures in Trendelenburg. Use of the lowest effective Trendelenburg angle and pneumoperitoneum pressure, avoidance of unnecessary prolongation of head down positioning, and continued awareness of the head and ears during lengthy procedures appear reasonable preventive measures [3, 11]. A limitation of our report is the absence of formal audiometric assessment and the incomplete availability of detailed anesthetic ventilatory parameters, which limits correlation between otologic findings and intraoperative pressure-related variables. Nevertheless, the temporal association with prolonged Trendelenburg positioning and pneumoperitoneum, together with the similarity to previously published reports, supports a probable procedure related mechanism [2–5, 10, 11]. Perioperative otorrhagia should therefore be recognized as an uncommon but relevant complication of robotic colorectal surgery that may occur even under moderate positioning conditions and is usually managed successfully with conservative treatment [3, 4, 10, 11].
